# Factors Associated with Intention to Receive Influenza and Tetanus, Diphtheria, and Acellular Pertussis (Tdap) Vaccines during Pregnancy: A Focus on Vaccine Hesitancy and Perceptions of Disease Severity and Vaccine Safety

**DOI:** 10.1371/currents.outbreaks.d37b61bceebae5a7a06d40a301cfa819

**Published:** 2015-02-25

**Authors:** Allison T. Chamberlain, Katherine Seib, Kevin A. Ault, Walter A. Orenstein, Paula M. Frew, Fauzia Malik, Marielysse Cortés, Pat Cota, Ellen A. S. Whitney, Lisa C. Flowers, Ruth L. Berkelman, Saad B. Omer

**Affiliations:** Department of Epidemiology, Rollins School of Public Health, Emory University, Atlanta, Georgia, USA; Department of Medicine, Division of Infectious Disease, Emory University, Atlanta, Georgia, USA; Department of Gynecology and Obstetrics, School of Medicine, Emory University, Atlanta, GA, USA; Department of Obstetrics and Gynecology, University of Kansas Medical Center, Kansas City, KS, USA; Department of Medicine, Division of Infectious Disease, Emory University, Atlanta, Georgia, USA; Emory Vaccine Center, Emory University, Atlanta, Georgia, USA; Department of Medicine, Division of Infectious Disease, Emory University, Atlanta, Georgia, USA; Department of Behavioral Sciences and Health Education, Rollins School of Public Health, Emory University, Atlanta, Georgia, USA; Department of Global Health, Rollins School of Public Health, Emory University, Atlanta, Georgia, USA; Department of Global Health, Rollins School of Public Health, Emory University, Atlanta, Georgia, USA; Georgia Obstetrical and Gynecological Society, Suwannee, Georgia, USA; Department of Epidemiology, Rollins School of Public Health, Emory University, Atlanta, Georgia, USA; Department of Gynecology and Obstetrics, Emory University, Atlanta, Georgia, USA; Department of Epidemiology, Rollins School of Public Health, Emory University, Atlanta, Georgia, USA; Department of Epidemiology, Rollins School of Public Health, Emory University, Atlanta, Georgia, USA; Emory Vaccine Center, Emory University, Atlanta, Georgia, USA; 6 Department of Global Health, Rollins School of Public Health, Emory University, Atlanta, Georgia, USA

**Keywords:** Influenza, maternal vaccination, pertussis, vaccination, vaccine hesitancy

## Abstract

BACKGROUND: Improving influenza and tetanus, diphtheria and acellular pertussis (Tdap) vaccine coverage among pregnant women is needed. PURPOSE: To assess factors associated with intention to receive influenza and/or Tdap vaccinations during pregnancy with a focus on perceptions of influenza and pertussis disease severity and influenza vaccine safety. METHODS: Participants were 325 pregnant women in Georgia recruited from December 2012 – April 2013 who had not yet received a 2012/2013 influenza vaccine or a Tdap vaccine while pregnant. Women completed a survey assessing influenza vaccination history, likelihood of receiving antenatal influenza and/or Tdap vaccines, and knowledge, attitudes and beliefs about influenza, pertussis, and their associated vaccines. RESULTS: Seventy-three percent and 81% of women believed influenza and pertussis, respectively, would be serious during pregnancy while 87% and 92% believed influenza and pertussis, respectively, would be serious to their infants. Perception of pertussis severity for their infant was strongly associated with an intention to receive a Tdap vaccine before delivery (p=0.004). Despite perceptions of disease severity for themselves and their infants, only 34% and 44% intended to receive antenatal influenza and Tdap vaccines, respectively. Forty-six percent had low perceptions of safety regarding the influenza vaccine during pregnancy, and compared to women who perceived the influenza vaccine as safe, women who perceived the vaccine as unsafe were less likely to intend to receive antenatal influenza (48% vs. 20%; p < 0.001) or Tdap (53% vs. 33%; p < 0.001) vaccinations. CONCLUSIONS: Results from this baseline survey suggest that while pregnant women who remain unvaccinated against influenza within the first three months of the putative influenza season may be aware of the risks influenza and pertussis pose to themselves and their infants, many remain reluctant to receive influenza and Tdap vaccines antenatally. To improve vaccine uptake in the obstetric setting, our findings support development of evidence-based vaccine promotion interventions which emphasize vaccine safety during pregnancy and mention disease severity in infancy.

## Related Articles

The article is part of the *PLOS Currents Outbreaks *"Vaccine Hesitancy Collection".

## Introduction

Respiratory infections like influenza and pertussis during pregnancy can pose serious risks to mother and infant.^1^^,^ 2 ^,^ 3 ^,^ 4 ^,^ 5 ^,^ 6 ^,^ 7 ^,^ 8 Pregnant women are at increased risk of complications from influenza, and infants are not recommended to receive an influenza vaccine until 6 months of age.^9^ For pertussis, infants under 2 months of age, prior to the recommended age for vaccination, have the highest rates of hospitalization and death.^10^ Antenatal vaccination against these diseases not only protects mothers, but studies have suggested protection can be conferred to infants through maternal-fetal transfer of antibodies through the placenta.^12^^,^ 13 Influenza vaccination during pregnancy can also protect against adverse fetal outcomes like preterm birth and small for gestational age as well as respiratory illnesses during infancy.^14^^,^ 15

Antenatal influenza vaccination recommendations have been in place since the 1960’s^16^ , and in the U.S., the Centers for Disease Control and Prevention (CDC) began recommending tetanus, diphtheria, and acellular pertussis (Tdap) vaccination during pregnancy, preferably in the third or late second trimester, in 2011.^17^ Based on previous research among pregnant women and healthy adults, both vaccines are considered safe during pregnancy.^18^^,^ 19 ^,^ 20 ^,^ 21 ^,^ 22 ^,^ 23 ^,^ 24 Despite CDC recommendations, coverage estimates for both vaccines remain suboptimal in the U.S. The influenza vaccine coverage rate estimated by CDC among pregnant women is the U.S. for the 2012 – 2013 season was 50.5%, and while coverage rates for antenatal Tdap vaccination are not yet available, estimates range between 2.6% - 10% (CDC, unpublished data, 2012).

Vaccinating pregnant women is a challenge. Studies exploring barriers to vaccinating women in the obstetric setting suggest that logistic barriers such as lack of storage space, knowledge gaps regarding vaccine safety or vaccine recommendations, and vaccine hesitancy all contribute to immunization decision-making.^25^ The aim of this descriptive analysis is to identify factors associated with an intention to receive influenza and/or Tdap vaccines during pregnancy among women who remained unvaccinated against influenza within the first three months (September – November) of the putative 2012/2013 influenza season in the U.S.

## Methods

Pregnant women included in these analyses were enrolled as part of a larger group-randomized trial entitled the “Emory MOMVAX study” to evaluate the effectiveness of a comprehensive, evidence-based vaccine education and promotion package on increasing antenatal influenza and Tdap vaccination in the obstetric setting. Women were recruited between December 11, 2012 and April 22, 2013 from 11 obstetric practices in Georgia participating in the Emory MOMVAX study. Recruiting women who remained unvaccinated against influenza by December likely increased the number of vaccine-hesitant women in our sample since women more likely to seek or accept vaccinations would have already received an influenza vaccination.

Following provision of informed consent, women were given a 28-item baseline survey in English to complete in the waiting area. These survey results are the focus of this paper. The survey included questions on demographics, influenza vaccine history, and knowledge, attitudes, and beliefs about influenza, pertussis and their accompanying vaccines during pregnancy. Perceptions of influenza vaccine safety were assessed through the level of agreement with the statement “Getting a flu vaccine while pregnant seems risky.” Perceptions of influenza and pertussis severity were assessed through the question “How serious do you think it would be if you got the following illnesses while pregnant?” Likewise, perceptions of influenza and pertussis severity during infancy were assessed through the question “After delivery, how serious do you think it would be if your newborn baby got the following illnesses within their first 6 months?” A team of clinicians, behavioral researchers, and communication specialists reviewed the questionnaire items to ensure clarity and adequacy of comprehension prior to administration.

Women were recruited by trained study personnel from the waiting areas of each participating practice. Eligibility criteria for participation were: being between 18 years and 50 years old, English-reading, currently pregnant, had not yet received a 2012 – 2013 seasonal influenza vaccine, and had not yet received a Tdap vaccine during their current pregnancy. After screening, written informed consent was obtained from each eligible woman interested in enrolling prior to administration of the baseline survey. While the intent was to complete the baseline survey prior to exposure to any intervention materials under evaluation in the MOMVAX study, if a woman was unable to finish the baseline survey prior to being called back for her scheduled appointment, she could complete the survey following her appointment. If, however, the woman returned to complete the baseline survey and indicated she had received an influenza and/or Tdap vaccine during her visit, she was no longer eligible for enrollment. At the time of enrollment and completion of the baseline survey, no attempts were made by the study personnel to provide any information about influenza, pertussis or their respective vaccinations.

The Institutional Review Boards of Emory University and the Medical Center of Central Georgia reviewed and approved this study. SAS version 9.3 statistical software (SAS Institute, Cary, NC) was used in 2013 for data analysis, including frequency calculations and proportion comparisons with chi-square and Fisher's exact tests. Women for whom survey data were missing on any given variable were retained in the denominator for univariate frequency calculations; missing data occurring in <1% of women were excluded from bivariate analyses, unless otherwise noted. Bivariate associations with a p-value < 0.05 were considered statistically significant.

## Results

One-thousand four-hundred and thirty-six women were screened between December 11, 2012 – April 22, 2013. [Figure 1]

**Schematic of study population included and excluded from baseline survey analyses.**
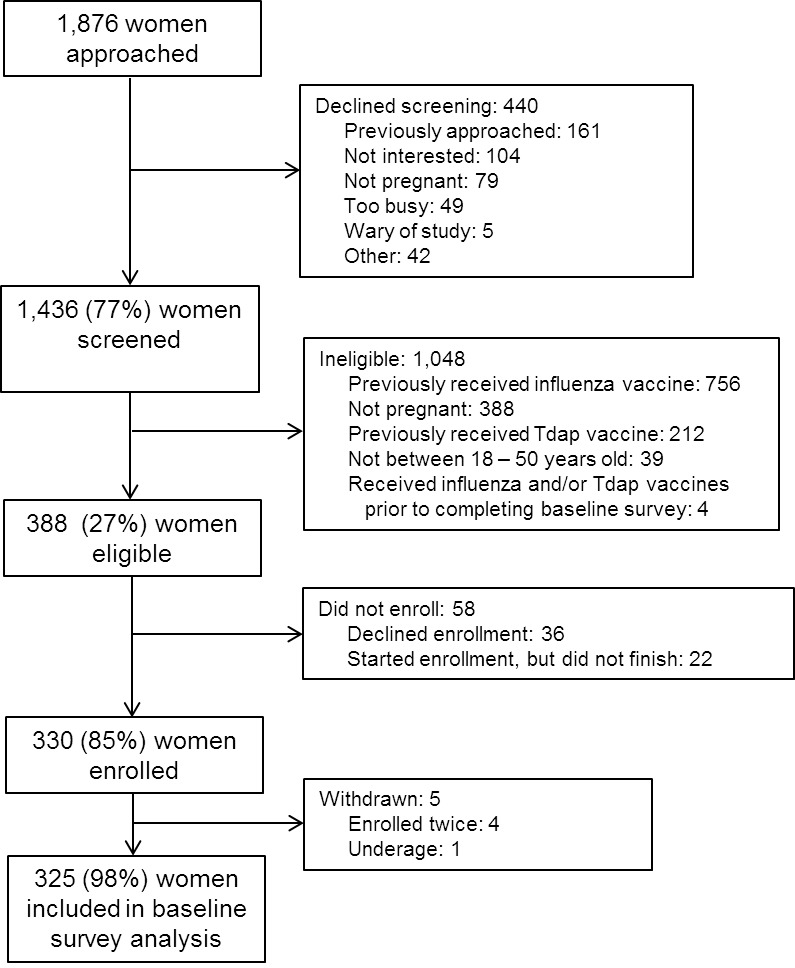

Three-hundred eighty-eight women were eligible, and 325 women were enrolled and completed the baseline survey. Among 1,037 pregnant women screened, 609 (59%) and 212 (20%) were ineligible because they had already received a 2012 – 2013 influenza vaccine or a Tdap vaccine, respectively. The mean age of participants was 27.2 years and the mean parity was 1.1 children. Approximately 47% of participants were Caucasian/White and 41% were African American/Black. [Table 1]

**Figure:**
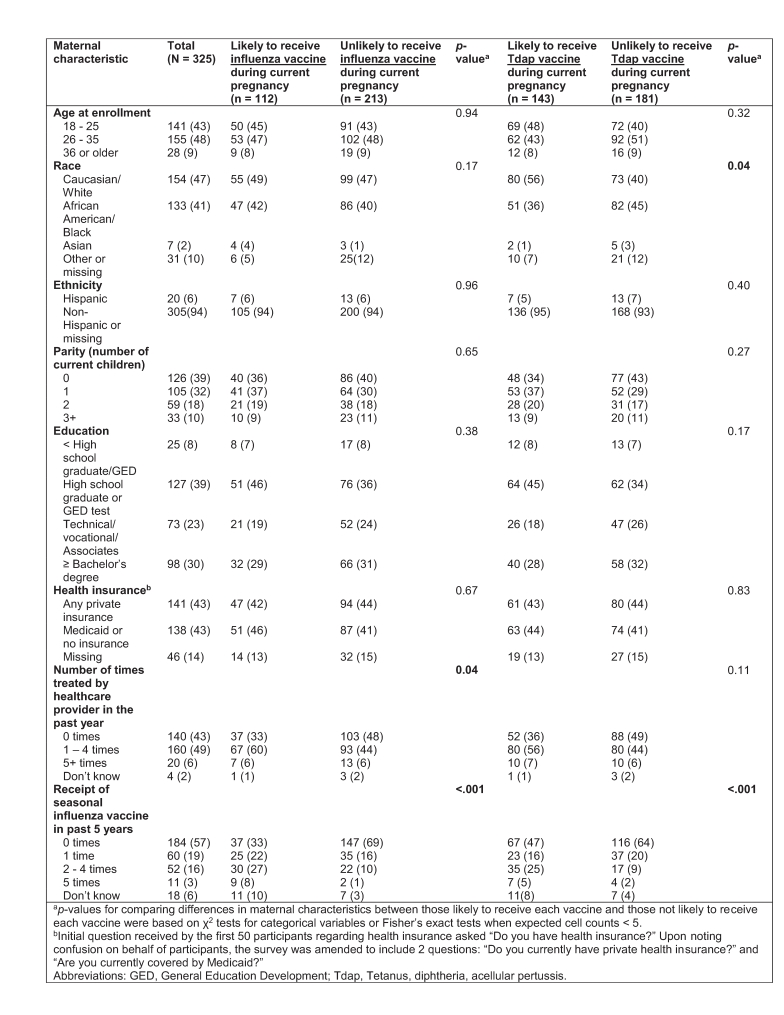
**Table 1: Maternal characteristics and associations with intention to receive antenatal influenza and Tdap vaccines**

The proportion of participants reporting at least some type of private health insurance was approximately equivalent to the proportion reporting no insurance or coverage only by Medicaid (43.4% vs. 42.5%).

More than half (57%) of the women reported not having received a seasonal influenza vaccine in the past five years, while another 19% reported having only received a seasonal influenza vaccine once in the past five years. [Table 1] Sixty percent of participants considered their OB/GYN their primary care physician, yet two-hundred sixteen (66%) reported never having received any type of vaccine in an OB/GYN doctor’s office. Thirty women (9%) reported having received a seasonal and/or H1N1 influenza vaccine in an OB/GYN’s office before. Over one quarter (26%) reported feeling hesitant (i.e. worried or concerned) about receiving vaccines recommended by their physician during pregnancy.

White women were significantly more likely to intend to receive a Tdap vaccine during their current pregnancy than women of other races, and intention to receive an influenza vaccine was significantly associated with the number of times treated by a healthcare provider in the past year. [Table 1] Intention to receive antenatal influenza and/or Tdap vaccines was also significantly associated with previous receipt of influenza vaccination in the past five years. There were no significant differences in proportions of women enrolled in control arm practices versus intervention arm practices on perceptions of disease severity during pregnancy, perceptions of disease severity for their newborn, intended likelihood of antenatal influenza vaccine receipt, intended likelihood of antenatal Tdap vaccine receipt, vaccine hesitancy, or perceptions of safety of influenza vaccination during pregnancy (data available upon request).

Two-hundred sixty five women (82%) agreed with the statement “Influenza is a concern for pregnant women,” and 238 (73%) believed influenza infection would be serious or very serious during pregnancy. Two-hundred sixty two (81%) believed contracting pertussis during pregnancy would be serious or very serious. Additionally, 87% and 92% believed influenza and pertussis, respectively, would be serious or very serious to their newborn within the first six months of life.

Despite perceptions of severity, only 112 (34%) and 143 (44%) reported they were likely to receive an influenza vaccine or Tdap vaccine, respectively, during their current pregnancy. [Figure 2]

**A). Perceived severity of influenza and pertussis during pregnancy and intention to get vaccinated during pregnancy. B). Perceived severity of influenza and pertussis during first 6 months of infancy and intention to get vaccinated during pregnance.**
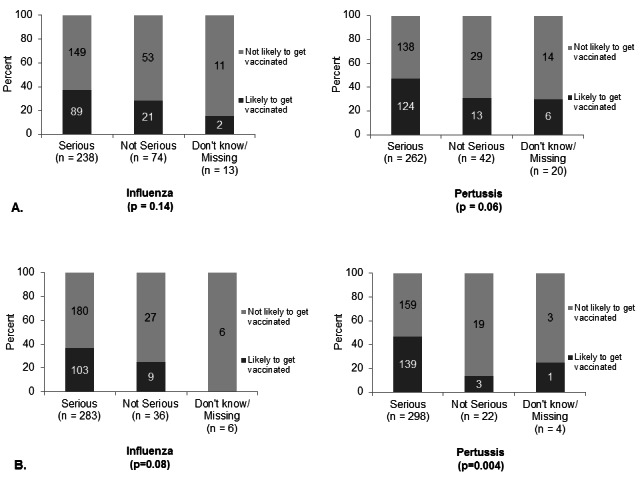

Perception of influenza disease severity for themselves or their newborns was not significantly associated with an intention to receive an influenza vaccine during pregnancy, but perception of pertussis severity for their infant was strongly associated with intention of antenatal Tdap vaccination (p=0.004). [Figure 2]

Regarding influenza vaccine safety, 149 women (46%) agreed with the statement “Getting an influenza vaccine while pregnant seems risky.” Compared to women who perceived the vaccine as safe, women who had low perceptions of influenza vaccine safety were significantly less likely to intend to receive an influenza vaccine (48% vs. 20%; p < 0.001) or a Tdap vaccine (53% vs. 33%; p < 0.001) during their current pregnancy. [Figure 3]

While a lower perception of influenza vaccine safety was associated with a higher probability of non-intention to be vaccinated, substantial proportions of women who perceived the influenza vaccine as safe still did not intend to be vaccinated (52% and 47% for influenza and Tdap, respectively). [Figure 3]

**Perception of safety of influenza vaccine during pregnancy and intention to receive influenza or Tdap vaccinations during pregnancy:**
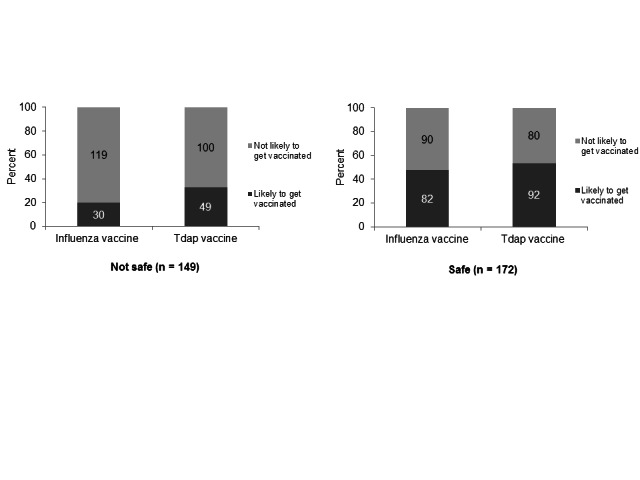

## Discussion

Antenatal vaccination against influenza and pertussis not only protects the mother from contracting these diseases, but it is also the first step towards protecting infants during their first 3 months of life.^26^ Efforts have been made in the U.S. by the American Congress of Obstetrics and Gynecology (ACOG) and other public health entities to stress the importance of influenza and Tdap vaccination during pregnancy.^27^^,^ 28 ^,^ 29 ^,^ 30 ^,^ 31 With nearly 60% of pregnant women screened for this study ineligible to enroll because they reported having already received a 2012 - 2013 influenza vaccine, efforts in promoting antenatal influenza vaccination have been successful. In contrast, this study suggests that among pregnant women who remain unvaccinated against influenza by December (when most women willing to get vaccinated probably would have already received the influenza vaccine), a hesitancy that surpasses general concerns about vaccine safety remains.

While results from this survey underscore findings from other studies which describe influenza vaccine hesitancy among pregnant women, the influenza-based findings presented here are juxtaposed with new insights on perceptions of Tdap vaccination during pregnancy.^25^^,^ 32 ^,^ 33 ^,^ 34 ^,^ 35 Most women enrolled in this study were aware of the dangers influenza and pertussis pose to themselves and their infants, yet over one-quarter indicated hesitancy about receiving any vaccines recommended during pregnancy. Nearly half of women perceived the influenza vaccine as unsafe during pregnancy, and more women were likely to receive a Tdap vaccine than an influenza vaccine for all levels of perceived disease severity for themselves. Since contracting influenza is more common and poses a greater threat to pregnant women than pertussis, it is concerning that more women perceived pertussis as more serious during pregnancy than influenza.^9^

Even though 60% of participants consider their OB/GYN to be their primary care physician, only one-third reported ever receiving a vaccine from their OB/GYN. These data mirror findings from other healthcare utilization studies and illuminate a gap in both service and expectation in the adult immunization system.^36^^,^ 37 Continuing to make vaccination a routine part of women’s health can help normalize vaccination within the obstetric setting.^38^ As obstetric healthcare providers become more accustomed to and comfortable with providing vaccines, women (pregnant or otherwise) will have greater access to and possibly acceptance of vaccines.

It is important to note the chronological context of this survey in relation to recent changes in antenatal Tdap recommendations in the U.S. While the U.S. Advisory Committee on Immunization Practices (ACIP) first recommended provision of Tdap at every pregnancy in October 2012, there were gaps between when this recommendation was made and when it was published.^39^^,^ 40 ^,^ 41 Since this survey was administered between December 2012 and April 2013, data were collected during the initial rollout of these new recommendations. By virtue of its timing, this survey provides a baseline assessment of pregnant women’s perceptions towards pertussis and Tdap in the U.S., thereby enabling changes in perceptions to be measured from this point forward.

This study has some important limitations. Since data were collected by self-report and not verified with medical records or vaccine registry data, there is potential for recall bias. Any recall bias which may have been introduced is assumed to have been non-differential with respect to characteristics likely to be associated with intention to receive antenatal influenza and/or Tdap vaccines. Additionally, while we excluded women who indicated having received an influenza and/or Tdap vaccine before completing her baseline survey, some women enrolled from intervention arm practices could have been exposed to the vaccine promotion materials under evaluation in the MOMVAX study prior to completing their baseline surveys. Since we did not find any significant differences between arms of the MOMVAX study on baseline measures of perceptions of disease severity, intended likelihood of vaccine receipt, vaccine hesitancy, or perceptions of safety of influenza vaccination during pregnancy, we do not believe limited exposure to promotional intervention materials related to the MOMVAX study prior to completion of the baseline survey had a differential impact on the women enrolled from intervention arm practices versus control arm practices. This study was also U.S.-based, so while results may be applicable to other countries, it may be important to replicate this type of survey among late-acceptors of antenatal influenza vaccines in other regions as well.

To further improve antenatal influenza vaccine coverage and to encourage antenatal Tdap vaccination, promotional efforts tailored specifically to late acceptors of influenza vaccination or vaccine-hesitant women is important. Other studies which have tested messaging techniques have started to emphasize this need to adapt messages based upon individuals’ preconceptions and attitudes towards vaccination.^42^ Since these results show that perceiving pertussis as serious for their infant is strongly associated with intention to receive an antenatal Tdap vaccine, explaining disease effects on infants may be an effective promotional strategy for women reluctant to receive vaccines. Continuing to promote, discuss, and offer influenza vaccine repeatedly and late into an influenza season is especially important for women who may be hesitant, but still interested in receiving an influenza vaccine. Likewise, continuing to discuss and promote Tdap vaccination throughout pregnancy can remind and encourage women to receive a Tdap vaccination before delivery. Since patient education on antenatal vaccination is likely to come from obstetricians, continuing to develop and evaluate nuanced tools for promoting influenza and Tdap vaccines during pregnancy is needed.

## Competing Interest

Allison Chamberlain, Katherine Seib, Paula Frew, Marielysse Cortés, Ellen Whitney, Ruth Berkelman, Saad Omer, Walter Orenstein, Fauzia Malik, Pat Cota and Lisa Flowers have no conflicts of interest to report. Kevin Ault has acted as a consultant on maternal immunization with the Centers for Disease Control and Prevention, the National Institute of Allergy and Infectious Diseases and the American College of Obstetricians and Gynecologists. He also serves on a data safety and monitoring committee with Novartis.
